# Molecular Mechanism of Ferroptosis in Orthopedic Diseases

**DOI:** 10.3390/cells11192979

**Published:** 2022-09-24

**Authors:** Lu Gao, Weizhong Hua, Lixiang Tian, Xuchang Zhou, Dongxue Wang, Yajing Yang, Guoxin Ni

**Affiliations:** 1 School of Sports Medicine and Rehabilitation, Beijing Sport University, Beijing 100084, China; 2 School of Physical Education, Shanghai University, Shanghai 200444, China; 3 National Cancer Center/National Clinical Research Center for Cancer/Cancer Hospital & Shenzhen Hospital, Chinese Academy of Medical Sciences and Peking Union Medical College, Shenzhen 518116, China

**Keywords:** ferroptosis, regulation of ferroptosis, bone marrow injury, osteoporosis, osteoarthritis, osteosarcoma, orthopedic disease

## Abstract

Ferroptosis is a new iron-dependent programmed cell death process that is directly mediated by the accumulation of lipid peroxides and reactive oxygen species. Numerous studies have shown that ferroptosis is important in regulating the occurrence and development of bone-related diseases, but the underlying mechanisms are not completely clear. Herein, we review the progress of the mechanism of ferroptosis in bone marrow injury, osteoporosis, osteoarthritis, and osteosarcoma and attempt to deeply understand the regulatory targets of ferroptosis, which will open up a new way for the prevention and treatment of orthopedic diseases.

## 1. Introduction

Programmed Cell Death (PCD) is an active and orderly process of cell death, which plays an important role in the evolution of living organisms, the stability of the internal environment, and the development of multiple systems [1]. PCD includes cell apoptosis, necrosis, pyroptosis, ferroptosis, and autophagy [2]. Apoptosis is characterized by nuclear fragmentation, the formation of apoptotic bodies, and the activation of proteins such as pro-apoptotic B cell lymphoma-2 (BCL-2). It leads to the increase of mitochondrial outer membrane permeabilization (MOMP) and the production of reactive oxygen species (ROS), thereby releasing apoptotic factors and promoting cysteine cascade activation [3,4]. During necrosis, the plasma membrane rupture, cytoplasmic organelle swelling, internucleosomal DNA fragmentation deficiency, ATP consumption, and damage-associated molecular patterns (DAMPs) release [5,6]. When pyroptosis occurs, the plasma membrane ruptures, the inflammation-related active IL-1β and IL-18 are released, and the caspase-1 and gasdermin D (GSDMD) are activated by hydrolysis [7,8]. Autophagy is characterized by the accumulation of autophagic vacuoles, cytoplasmic vacuolation, non-condensation of chromatin, the transformation of microtubule-associated protein light chain 3 (LC3)-I to LC3-II, and p62-cleaved [9,10]. 

Iron is a trace element with redox activity in natural organisms, which is involved in the regulation of cell survival and death. Mammalian cells require enough iron to maintain enzyme activity, lipid metabolism, and protein synthesis [11]. Under pathological conditions, the accumulation of excessive iron and ROS can lead to cell death, that is, ferroptosis. The concept of ferroptosis was proposed by Scott J Dixon’s team in 2012. Ferroptosis is an iron-dependent, novel PCD mode different from apoptosis, necrosis, and autophagy [12]. In terms of cell morphological characteristics, mitochondria atrophy, mitochondrial crisis decrease or even disappear, and membrane density increase in cells undergoing ferroptosis. In terms of biological characteristics, cystine uptake decreased, glutathione (GSH) depletion, inhibition of System X_c_^-^ activity, and abnormal aggregation of iron ions and ROS. At the gene level, ferroptosis is mainly regulated by ribosomal protein L8 (RPL8), iron-responsive element-binding protein 2 (IREB2), acyl-CoA synthetase family member 2 (ACSF2), as well as the regulation of the metabolic and storage genes TFRC, ISCU, FTH1, FTL, and SLC11A2 [13].

**Figure 1:**
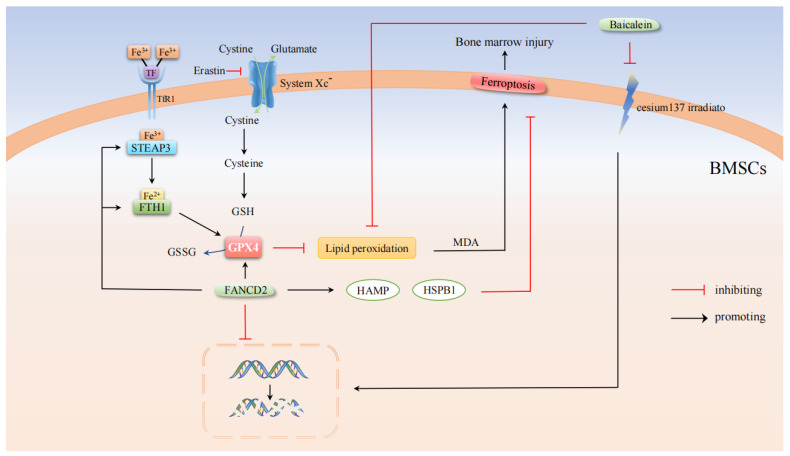
Molecular pathways in the regulation of bone marrow injury by ferroptosis. FANCD2 alleviated ferroptosis and reduced DNA damage by down-regulating Fe^2+^ levels and up-regulating GPX4 expression. Baicalein, the ferroptosis inhibitor, alleviated FA caused by radiation-induced DNA break repair defects, thereby alleviating bone marrow injury.

The occurrence and development of ferroptosis depend on the interaction of amino acids, lipids, and iron metabolism. Cystine/GSH/GPX4 is a classical ferroptosis inhibition system. System X_c_^-^ is an amino acid transporter composed of the transmembrane protein complex SLC7A11 and SLC3A2 subunits, in which SLC7A11 is highly specific for cystine and glutamate, which is essential for GSH synthesis. The GSH pathway has been identified as a key antioxidant defense pathway, so one approach to induce ferroptosis is to directly use compound proteins that inhibit System X_c_^-^ function. The commonly used inducer of ferroptosis is Erastian. The polyunsaturated fatty acids (PUFAs) treated by Erastin undergo a Fenton reaction with reduced Fe^2+^ under the action of a large amount of ROS to produce lipid peroxide (LPO) [14]. LPO is further reacted to produce toxic substances such as Malondialdehyde (MDA) and 4-Hydroxynonenal (4-HNE) [15]. Glutathione peroxidase 4(GPX4) is an antioxidant that can convert reduced GSH to oxidized GSSH, eliminate LPO and protect the integrity of cell membrane (Figure 1). Conversely, if GPX4 is inhibited, PUFAs will be oxidized, thus leading to the occurrence of ferroptosis [16,17]. Therefore, the induction of ferroptosis in cancer cells by inhibiting GPX4 is currently promising as a therapeutic strategy for some cancers. In summary, Cystine/GSH/GPX4 pathway can inhibit ferroptosis and is an important regulatory mechanism of ferroptosis.

Studies have found that the positive regulation of ferroptosis can be programmed to kill cancer cells, thus providing a potential approach for cancer treatment [18,19]. On the contrary, its negative regulation can inhibit cell death, thus improving the feasibility of treating ferroptosis-related diseases, such as the prevention of neurodegenerative diseases [20,21]. Recent studies have shown that ferroptosis has become a popular research object in bone-related diseases. Song et al. [22] demonstrated for the first time that FANCD2, a nuclear protein involved in DNA damage repair, could prevent bone marrow injury mediated by ferroptosis. In type 2 diabetic osteoporosis, melatonin inhibits high glucose-induced osteoblast ferroptosis by activating Nrf2/HO-1 signaling pathway [23]. D-mannose inhibits chondrocyte ferroptosis in a HIF-2α-dependent manner, thereby slowing down the progression of osteoarthritis [24]. Studies have also found that β-phenethyl Isothiocyanate induced apoptosis signal transduction in human osteosarcoma cells by changing iron metabolism, disrupting the redox balance, and activating the MAPK signaling pathway provides an effective strategy for osteosarcoma [25]. However, research has found that Erastin-induced ferroptosis altered the blood index values, causing mild cerebral infarction of the brain and enlarged glomerular volume of the kidney [26]. It can be concluded that induction of ferroptosis in cancer cells may cause pathological changes in healthy tissues and have certain toxic side effects. Considerable evidence supports the role of ferroptosis in the pathogenesis of orthopedic diseases. Therefore, the regulation of ferroptosis in bone-related cells may be a potential target for the treatment of the above orthopedic diseases.

In this review, we provide a brief overview of the classical pathways of ferroptosis and describe the already existing and proposed therapeutic strategies for bone marrow injury, osteoporosis, osteoarthritis, and osteosarcoma to target key regulators of the ferroptosis pathway for orthopedic-related diseases. An in-depth investigation of the molecular regulatory mechanism of the ferroptosis in orthopedic diseases will provide a theoretical basis for the prevention and treatment of such diseases.

## 2. Bone Marrow Injury

Bone marrow is the hematopoietic tissue of the human body, in which red bone marrow can produce red blood cells, platelets, and various white blood cells. Platelets have hemostatic effect, and white blood cells can kill and inhibit various pathogens, including bacteria and viruses, etc. [27]. At present, the regulation of various types of cell death in tumor cells is a popular target for new antitumor drugs under development [28,29]. Bone marrow injury is one of the most common dose-limiting side effects of such drugs, which may lead to neutropenia, thrombocytopenia or anemia, etc., thus further causing drug dose reduction, treatment time delay, and efficacy reduction in cancer treatment [22]. Studies have found that Fanconi anemia complementation group D2 (FANCD2), a nuclear protein involved in DNA damage repair, can inhibit damage in bone marrow stromal cells (BMSCs) with hematopoietic reconstitution and immune modulation caused by ferroptosis [22]. FANCD2 mono-ubiquitination is the most important step in the DNA cross-linking repair pathway. This study found that Erastin-induced ferroptosis significantly increased the level of mono-ubiquitinated FANCD2, thereby limiting DNA damage in BMSCs. In BMSCs with FANCD2 knockdown, Fe^2+^ and MDA levels increased, and GSH consumption increased. The mRNA expression of FTH1 (an inhibitor of ferroptosis by binding Fe^2+^ [30]) and STEAP3 (a metal reductase capable of converting Fe^3+^ to Fe^2+^) is inhibited. Similarly, the mRNA expression of GPX4 was downregulated, inducing the downregulation of the basic negative regulators of ferroptosis of HAMP [31] and HSPB1 [32] and increased DNA damage. In brief, FANCD2 reduces the side effects of anticancer drugs on bone marrow by regulating ferroptosis. Clinical studies have shown that bone marrow hematopoietic failure is the main cause of death in patients with Fanconi anemia (FA), a disease associated with one or more gene defects of 26 genes related to DNA double-strand break repair proteins [33,34,35]. When DNA is affected by radiation, chromosomes break and recombine, which leads to DNA breakage, and the FA protein repair mechanism is activated by the body. After 24 h of total body irradiation (TBI) of 8.5 Gy to bone marrow stromal cell lines of wild-type C57BL/6J mice, researchers injected the radiation alleviating drugs GS-nitroxide JP4-039 (anti-apoptosis), necrostatin-1 (anti-necrosis) and baicalein (anti-ferroptosis). The results showed that three drugs all improved the survival rate after radiation and had significant radioprotective effects, facilitated the repair of DNA damage, and thus slowed down bone marrow failure [36].

In summary, reducing the side effects of new anticancer drugs on bone marrow can be achieved by inhibiting the accumulation of iron and LPO in the process of ferroptosis by FANCD2 (Figure 1). The recently discovered Acyl-CoA synthetase long-chain family member 4 (ACSL4) is required for the synthesis of 5-hydroxyeicosatetraenoic acid (5-HETE), which can further induce ferroptosis [37]. However, whether FANCD2 is involved in the occurrence of ferroptosis by regulating the ACSL4-5-HETE pathway remains to be further investigated. Baicalein, a ferroptosis inhibitor, can alleviate the FA caused by radiation-induced DNA break repair defects and thus reduces bone marrow failure. At the same time, the same drug is relatively ineffective in FA mouse cell lines. Therefore, further studies are needed to explore the mechanism by which ferroptosis inhibitors alleviate radiation toxicity in FA mouse models.

## 3. Osteoporosis

With the aging of the global population, the incidence of osteoporosis (OP) is also increasing. OP is a systemic bone disease characterized by a decrease in bone density and quality, the destruction of bone microstructure, and an increase in bone fragility [38]. OP is divided into two major categories: primary-osteoporosis (POP) and secondary OP. POP can be divided into postmenopausal- osteoporosis (PMOP), senile-osteoporosis (SOP), and idiopathic OP. Moreover, OP caused by many diseases is called secondary OP, such as type 2 diabetic osteoporosis (T2DOP) [39]. OP occurs when the balance of bone remodeling is destroyed. The mechanisms of the balance destruction of bone remodeling include the following: (1) Iron overload interferes with the differentiation of stem cells into osteoblasts, which leads to the weakening of osteoblast function and further imbalance of bone metabolism in vivo, thus leading to OP; (2) Under the stimulation of iron overload, osteoclast bone resorption is enhanced, and bone loss exceeds the formation of new bone. Such excessive osteoclast activation will lead to bone loss, even pathological fracture [40,41,42]. Thus, iron overload is closely related to OP. Therefore, it is important to determine whether ferroptosis is involved in the pathogenesis of OP and to find more alternative treatments with osteoprotective effects.

### 3.1. OP-Associated Osteoblasts with Ferroptosis

The existence of iron homeostasis is important for the normal physiological function of cells. Studies have found that OP is closely related to iron homeostasis, and intracellular iron homeostasis is mainly regulated by a variety of iron-related proteins, including intracellular ferritin, divalent metal transporters 1 (DMT1), and transferrin receptors (TfR) and transferrin (Tf) [43]. Iron overload is caused by the overexpression of DMT1 in osteoblasts, resulting in oxidative stress response and ultimately affecting the osteogenic function of osteoblasts, which plays an important role in the pathogenesis of T2DOP [44,45,46]. Ma et al. [23] and Wang et al. [47] both found that high glucose (HG) could affect the mitochondrial morphology of osteoblasts by increasing the density of the bilayer mitochondrial membrane and decreasing the mitochondrial cristae and that these changes in mitochondria morphology are consistent with ferroptosis characteristics. HG also changes osteogenic function-related indicators, including decreased expression of osteoprotegerin (OPG) and osteocalcin (OCN), decreased activity of alkaline phosphatase (ALP), and reduced the formation of mineralized nodules [23,47]. In addition, HG can also increase the intracellular ROS content, promote the accumulation of LPO, and reduce the expression level of ferroptosis-related protein GPX4 in cells, leading to ferroptosis and osteogenic function decline in osteoblasts. Previous studies have confirmed that nuclear factor erythroid 2-related factor 2 (Nrf2) and heme oxygenase-1 (HO-1) signaling pathways are directly downstream of ROS. It is involved in the regulation of the transcription of antioxidant-responsive element (ARE)-dependent genes to balance oxidative mediators and maintain cellular redox homeostasis [48] and is therefore also considered to be an important regulator of ferroptosis [49,50]. It was found that endogenous antioxidant Melatonin (N-acetyl-5-methoxytryptamine) reduced the levels of LPO and ROS in MC3T3-E1 cells exposed to HG (25.5 mM) by activating the Nrf2/HO-1 signaling pathway, increased activity of GPX4 and SLC7A11, and inhibited HG-induced ferroptosis. This, in turn, enhanced osteogenic ability, promoting trabecular bone formation, increasing bone mineral density (BMD) and bone volume relative to total tissue volume (BV/TV) and trabecular number (Tb.N), and improving bone microstructure. Knockdown of Nrf2 by siRNA inhibited the beneficial effect of melatonin on osteoblast activity. Further studies showed that the Nrf2 signaling pathway played a critical role in the intervention of melatonin on OP [23]. It has also been found that one of the most common causes of surgical failure and revision in total hip arthroplasty (THA) is aseptic loosening (AL). AL may be caused by particles induced osteolysis (PIO) released from the implant surface, leading to programmed death of MC3T3-E1 osteoblasts. CoCrMo radionuclides (CoNPs) promote the ferroptosis of osteoblasts by down-regulating the NRF2-ARE signaling pathway and inducing peri-implant PIO. Therefore, the blockade of ferroptosis with ferrostatin-1 and Nrf2 activator Oltipraz significantly improves PIO induced by the implantation of particles, which provides a potential strategy for treating AL [51]. Mitochondrial ferritin (FtMt) is a protein that stores iron ions and has ferroxidase activity. It can reduce the amount of free Fe^2+^ in mitochondria and prevent excessive Fe^2+^ from occurring Fenton reaction, thereby reducing the content of ROS [52]. Wang et al. [47] found that under HG conditions, the increased expression of mitochondrial DMT1 in hFOB1.19 osteoblasts leads to iron overload, and the overexpression of FtMt can reduce the intracellular ROS level and inhibit ferroptosis in osteoblasts. However, the silenced FtMt induced the mitochondrial phagocytosis through ROS/PINK1/Parkin pathway, which promoted the ferroptosis of osteoblasts and accelerated the pathological process of OP.

**Figure 2:**
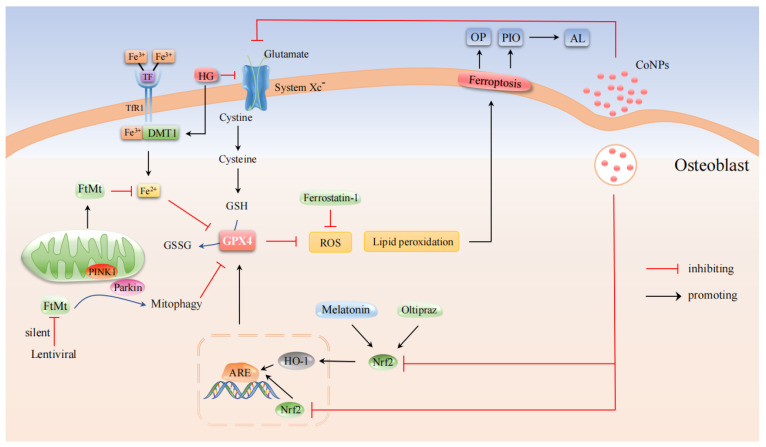
Molecular pathways in the regulation of OP-associated osteoblasts by ferroptosis. Melatonin inhibited osteoblast ferroptosis by activating Nrf2/HO-1 signaling and increasing GPX4 and SLC7A11 activity. Blockade of ferroptosis by the Nrf2 activator Oltipraz significantly ameliorated implanted particle-induced PIO. FtMt attenuated osteoblast ferroptosis by reducing the amount of free Fe^2+^ in the mitochondria.

In summary, inhibition of ferroptosis in osteoblasts slowed the development of OP (Figure 2). Melatonin can significantly reduce the level of ferroptosis through the activation of the Nrf2/HO-1 pathway, thereby improving the osteogenic ability of MC3T3-E1. Down-regulation of the NRF2-ARE signaling pathway by CoNPs can promote ferroptosis in osteoblasts, which are potential mechanisms affecting PIO. The combination of ferrostatin-1 and Oltipraz significantly improved osteoblast ferroptosis and PIO severity and provided an effective therapeutic strategy for the treatment of AL. FtMt can inhibit the occurrence of ferroptosis in osteoblasts and restore osteogenic function by reducing oxidative stress caused by excessive Fe^2+^, while FtMt deficiency induces mitochondrial phagocytosis through ROS/PINK1/Parkin pathway to cause ferroptosis in osteoblasts, thus leading to T2DOP. These results suggest that FtMt may be a potential target for T2DOP treatment.

### 3.2. OP-Associated Osteoclasts with Ferroptosis

Ferritin (FTH) is a protein that stores excess cellular iron and may be degraded when cells are in an iron-deficient state, called “ferritin autophagy,” which will increase the sensitivity to ferroptosis caused by intracellular Fe^2+^ [53]. Osteoclasts, as acid-secreting cells and resorptive bone cells, contain abundant mitochondria to maintain high energy demand [54]. Therefore, mature osteoclasts require more intracellular free iron than other osteocytes [55]. Studies have found that ferritin autophagy is a process initiated by the degradation of ferritin heavy chain-nuclear receptor Coactivator 4 (FTH-NCOA4) complex autophagosome. It is activated by the receptor activator of nuclear factor Kappa B ligand (RANKL) under normoxic conditions. The occurrence of an iron deficiency response (increased TfR 1, decreased FTH) significantly increased MDA, and Fe^2+^ content decreased GSH level and induced osteoclast ferroptosis. In contrast, hypoxia-inducible factor 1 alpha (HIF-1α) can reduce RANKL-induced ferritin autophagy and autophagy flux under hypoxia and protect osteoclasts from ferroptosis. In vivo studies further showed that 2-methoxyestradiol (2ME2), the HIF-1α specific inhibitor, prevented bone loss in ovariectomized (OVX) mice [56]. Studies have also found that Artemisinin (ARS) and its related compounds are not only being used clinically as antimalarial drugs but also as a potential alternative drug in the treatment of bone loss. The possible mechanism is that ARS compounds significantly increase TFR1-mediated iron uptake during osteoclast differentiation. In the acidic endosome, Fe^3+^ is reduced to Fe^2+^, which is released into the cytoplasm as the liable iron pool (LIP) through DMT1. In addition, ARS compounds selectively inhibit osteoclast differentiation by downregulation of pathways involved in RANKL-induced osteoclastogenesis, resulting in the ferroptosis of osteoclasts [57].

**Figure 3:**
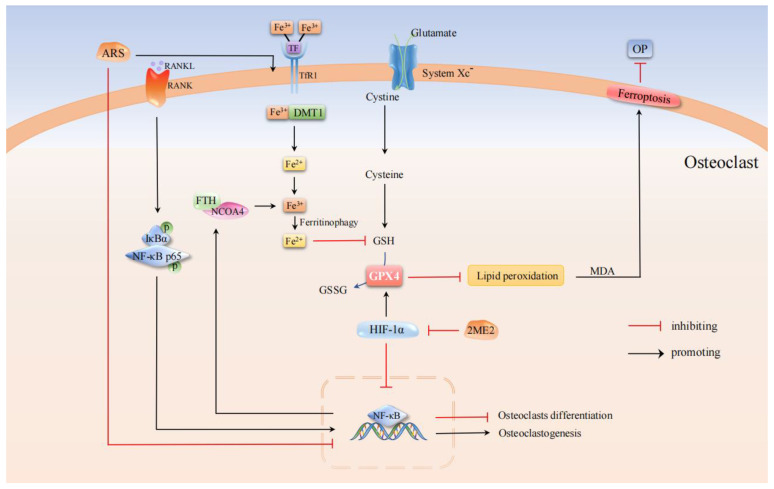
Molecular pathways in the regulation of OP-associated osteoclasts by ferroptosis. 2ME2, the HIF-1α specific inhibitor, induced osteoclast ferroptosis by increasing MDA and Fe^2+^ content through the RANKL pathway. ARS and its compounds down-regulated the RANKL-induced osteoclast generation pathway and significantly increased TFR1-mediated iron uptake to induce osteoclast ferroptosis.

In summary, ARS and its related compounds at non-cytotoxic concentrations significantly inhibited RANKL-induced differentiation of bone marrow-derived macrophages into osteoclasts, whereas 2ME2 induced ferritin autophagy by upregulating RANKL. Both lead to ferroptosis in osteoclasts, reduce osteoclast generation, and inhibit bone resorption, which may be an alternative treatment for OP (Figure 3). Although ARS was originally separated from Artemisia annua L and has a long history of safe application in malaria treatment, it remains unclear whether ARS compounds have side effects such as hypocalcemia. Further studies are needed to evaluate possible interactions with other anti-osteoporosis drugs currently used to ensure the efficacy and safety of ARS.

## 4. Osteoarthritis

Osteoarthritis (OA) is a common chronic degenerative joint disease affected by multiple factors such as heredity, age, gender, trauma, and biomechanical abnormalities [58,59]. It is mainly characterized by cartilage degeneration, synovial inflammation, and subchondral bone remodeling [60,61]. Articular cartilage, which is composed of chondrocytes, is essential for maintaining the integrity of the extracellular matrix [62] and balancing articular cartilage homeostasis to slow the progression of OA [63]. Current pharmacological treatment strategies for OA mainly focus on relieving clinical pain symptoms, improving joint function, and improving bone mass but usually do not prevent disease progression [64]. Recent studies have found that chondrocyte ferroptosis is one of the causes of the progressive reduction of articular chondrocytes during OA progression. Yao et al. [64] used Interleukin-1 Beta (IL-1β) to simulate the inflammatory response and used ferric ammonium citrate (FAC) to simulate in vitro iron overload. These two measures all induced changes in ROS, lipid ROS accumulation, and ferroptosis-related protein expression in chondrocytes. It is manifested as promoting the expression of matrix metalloproteinase13 (MMP13) and inhibiting the expression of type II collagen (collagen II) in chondrocytes. It then disrupted the homeostasis of the chondrocyte matrix. Ferrostatin-1 is a ferroptosis-specific inhibitor that attenuates the ferroptosis-related ROS and protein expression changes induced by IL-1β and FAC, promoting the activation of the Nrf2 antioxidant system. In addition, this study used a surgically induced mouse model of destabilized medial meniscus (DMM) to induce OA in vivo. In addition, an intra-articular injection of Ferrostatin-1 rescued the expression of collagen II and GPX4 in the mouse OA model, inhibited the occurrence of ferroptosis in chondrocytes, and reduced cartilage degradation and OA progression. Hypoxia-inducible factor 2 alpha (HIF-2α) has been reported to play an essential role in cartilage development, OA progression, and sensitizing cells to ferroptosis [65,66,67]. There were in vitro studies using IL-1β to treat chondrocytes in the OA microenvironment. The result showed that, with the IL-1β stimulation, the HIF-2α and LPO levels were upregulated in the chondrocytes, and the GSH level was downregulated. However, upon treatment with D-mannose, a C-2 epimer of glucose, the changes in the LPO and GSH levels can be reversed. At the same time, the level of malondialdehyde (MDA), a byproduct of LPO, was also correspondingly reduced. It also elevated the RNA and protein levels of two key ferroptosis repressors, GPX4 and SLC7A11. Thus, it reduced the sensitivity of chondrocytes to ferroptosis [24]. In studies of in vivo anterior cruciate ligament transection (ACLT) -induced OA mouse models, D-mannose delayed the progression of OA in mice by inhibiting HIF-2α-mediated chondrocyte sensitivity to ferroptosis [24]. Moreover, during OA, the synovium develops interstitial vascularization, fibrosis, and hyperplasia, leading to synovitis [68], which is closely associated with joint dysfunction, injury, and pain. These factors also lead to cartilage degeneration in OA [69]. It has been found that Icariin (ICA) suppresses the effect of ferroptosis activator RSL3 on cell viability, LPO, iron content, and related protein expression in synovial cells, thus protecting LPS-induced synoviocytes from ferroptosis [70]. Therefore, this study provides a new strategy for the treatment of synovitis.

**Figure 4:**
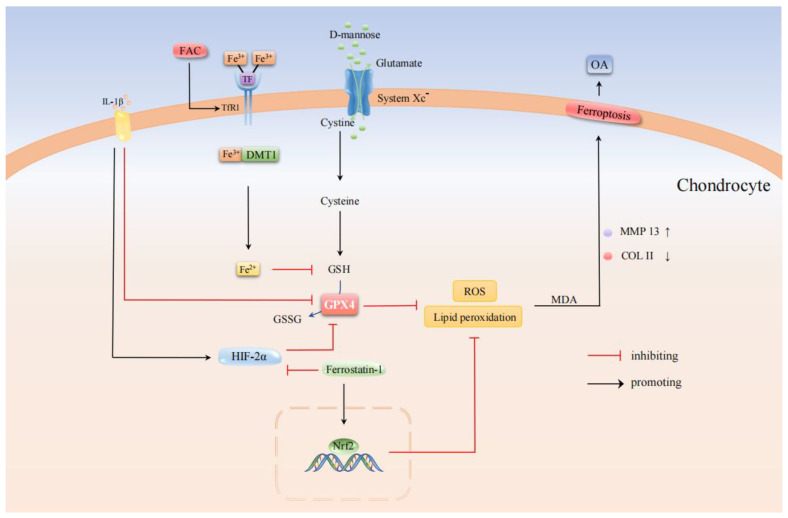
Molecular pathways in the regulation of osteoarthritis by ferroptosis. Ferrostatin-1 inhibited chondrocyte ferroptosis and slowed down the development of OA in mice by increasing the expression of collagen type II and GPX4. D-mannose reduces the sensitivity of chondrocytes to ferroptosis by increasing HIF-2α-mediated RNA and protein levels of GPX4 and SLC7A11.

In summary, chondrocytes undergo ferroptosis under inflammatory and iron overload conditions. At the same time, intra-articular injection of Ferrostatin-1 and oral D-mannose protected the articular cartilage and inhibited ferroptosis of synovial cells with ICA, all of which are important for alleviating the progression of OA (Figure 4). Notably, D-mannose mainly inhibited the catabolism of chondrocytes and downregulated MMP13 levels in vivo, thus maintaining COL2A1 expression, which varies from the results of previous studies. It may be caused by methodological differences because many factors affect the biological effects of D-mannose, such as the pathogenic mechanism, cell sensitivity, administration method, and dose of OA, which need to be considered comprehensively.

## 5. Osteosarcoma

Osteosarcoma (OS) is the most common primary malignant bone tumor [71,72], in adolescents, with a highly aggressive and distal metastatic [73]. However, conventional adjuvant chemotherapy regimens have reached a bottleneck. Cisplatin is a common chemotherapeutic agent for treating osteosarcoma, causing relatively few side effects. Nevertheless, OS patients often develop cisplatin resistance after long-term use, leading to treatment failure [74]. Therefore, inhibition of resistance to cisplatin in OS patients yields better therapeutic effects. The signal transducer and activator of transcription 3 (STAT3) belongs to the STAT family and is an important transcription factor involved in inflammation and tumor progression [75]. Nrf2 is a transcription factor that regulates the expression of various antioxidant proteins. STAT3 enhances the antioxidant capacity of cells by activating Nrf2, thus protecting cells from oxidative damage [76]. Studies of incubating OS cells (MG63 and Saos2) with increased doses of cisplatin have produced human osteosarcoma cells with cisplatin-resistant cells MG63/DDP and Saos2/DDP with increased viability and decreased mortality. It was also accompanied by decreased pSTAT3, Nrf2, and GPX4 protein levels and increased ROS, LPO, and MDA levels, causing ferroptosis and increased sensitivity to cisplatin [77]. Thus, long-term cisplatin induced the production of resistant osteosarcoma cells by inhibiting ferroptosis. STAT3/Nrf2/GPX4 signaling plays an important role in the drug resistance process of OS cells, which may be a potential therapeutic target to overcome cisplatin resistance in OS cells. Flavonoids have multiple biological functions, especially the anticancer effect induced by pro-oxidative activity [78]. Bavachin, a flavonoid, which Luo et al. [79] found to alter mitochondrial morphology in OS cells, resulting in smaller mitochondria, increased mitochondrial membrane density, and decreased mitochondrial cristae. Further studies revealed that Bavachin could upregulate the expression of transferrin receptors, such as DMT1 and P53, and downregulate the expression of ferritin light chain, ferritin heavy chain, p-STAT3, SLC7A11, and GPX4 in OS cells. Among these, P53 acts as an upstream mediator of SLC7A11, thus initiating ferroptosis in tumor cells [80]. More importantly, STAT3 overexpression, SLC7A11 overexpression, and pretreatment with pifithrin-α (P53 inhibitor) rescued Bavachin-induced ferroptosis in OS cells. In a word, the results indicate that Bavachin induces ferroptosis in OS cells by inhibiting the STAT3/P53/SLC7A11 axis [79]. The Mitogen-activated protein kinases (MAPK) pathway plays a crucial role in the signal transduction process of eukaryotic cells [81], as a key pathway in the OS-related mechanism, and helps to balance the relationship between tumor cell viability and mortality [82]. Phenethyl isothiocyanate (PEITC) can induce tumor cell cycle arrest and apoptotic [83]. Studies have found that PEITC can reduce the viability of human OS cells, inhibit cell proliferation, cause G2/M cell cycle arrest, alter iron metabolism, induce GSH depletion, generate ROS, activate the MAPK signaling pathway, and trigger ferroptosis in OS cells. Furthermore, PEITC with a dose of 30 mg/kg also significantly delayed tumor growth in the transplanted OS mouse model [25]. Tirapazamine (TPZ) is a hypoxic prodrug with antitumor effects in the tumor hypoxic microenvironment [84]. Shi et al. [85] demonstrated for the first time that TPZ induced ferroptosis in OS cells by inhibiting the expression of SLC7A11 under hypoxia conditions, thus preventing the proliferation and migration of osteosarcoma cells. EF24, a synthetic analog of curcumin, acts as an antitumor compound that induces apoptosis and inhibits proliferation and metastasis [86,87,88]. It has been shown that EF24 inhibited GPX4 expression and induced OS cells by increasing MDA levels, ROS levels, and intracellular iron ion levels, upregulating the expression of the cytoprotective enzyme heme oxygenase 1 (heme oxygenase 1, HMOX1), and induced ferroptosis in OS cells [89]. Therefore, EF24 may serve as a potential therapeutic agent for patients with HMOX1-positive OS.

**Figure 5:**
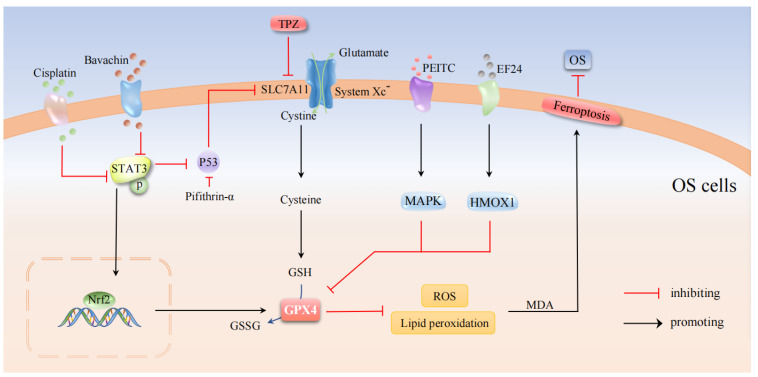
Molecular pathways in the regulation of osteosarcoma by ferroptosis. The anticancer drug cisplatin-induced ferroptosis by reducing STAT3 levels and increased the sensitivity of OS cells to cisplatin by inhibiting STAT3, while Bavachin also triggered OS cell ferroptosis and prevented OS cell spread by inhibiting STAT3 expression. PEITC and EF24 activated MAPK and HMOX1 signaling pathways, respectively, which can increase the lipid peroxidation level, intracellular iron concentration, and ROS level of human OS cells, thus inducing ferroptosis. TPZ induced ferroptosis in OS cells by inhibiting the expression of SLC7A11 in the classical ferroptosis signaling pathway.

In summary, we suggest that cisplatin, Bavachin, PEITC, EF24, and TPZ all show different degrees of cancer inhibition by inducing ferroptosis in OS cells (Figure 5). It has been demonstrated that radiotherapy can regulate ferroptosis in multiple cancer cells through pathways such as downregulating SLC7A11 and upregulating ACSL4. The impact of ferroptosis in the non-pharmacological treatment of OS remains to be demonstrated due to the lack of adequate evidence-based practice.

## 6. Conclusions and Prospects

The dynamic balance of iron content is essential for human activity and is regulated through multiple pathways. Ferroptosis is a kind of iron-dependent PCD process, while cell apoptosis, autophagy, necrosis, and other death mode is quite different. It is mainly induced by iron-dependent LPO and ROS, which lead to cell membrane rupture, and minor mitochondrial damage occurs. Its remarkable features were iron and ROS aggregation and a series of abnormal gene expressions and signal transduction. The regulation of ferroptosis can be initiated through the activation of transferrin (Tf) in an extrinsic pathway. Ferroptosis can also be regulated without affecting iron homeostasis in the organism by activating GPX4 or inhibiting the cell membrane transporter System X_c_^-^. A growing number of studies have proved that the development of orthopedic diseases is closely related to ferroptosis and may delay the course of the disease in the following two ways. (1) Negative regulation of ferroptosis limits cytotoxic LPO from protecting cells from iron deposition, such as inhibiting the death of BMSCs with hematopoietic reconstruction and immune regulation function, preventing bone marrow injury. This can prevent the ferroptosis of osteoblasts from preventing bone loss, improving bone microstructure, and treating OP, and can inhibit the ferroptosis of chondrocytes, restore normal metabolism, synthesis and self-repair function, and delay OA progression. (2) Positively regulate ferroptosis, induce ferroptosis in osteoclasts, reduce osteoclasts oogenesis, and inhibit bone resorption from preventing OA; and can also be used to kill OS cells, thus providing a potential treatment for OS. Therefore, novel ferroptosis inhibitors and activators have become popular research subjects in cell biology and chemical biology, providing new strategies for the targeted treatment of orthopedic diseases. However, the understanding and exploration of iron metabolism disorder and ferroptosis under the pathological state of bone marrow injury, OP, OA, and OS is still in its initial stage. Many important scientific problems need to be solved urgently, and new prevention and control strategies need to be explored deeply.

- Research on activators and inhibitors regulating key targets of ferroptosis.
- Research on interventions (DNA repair proteins, flavonoids, antineoplastic agents, etc.) of ferroptosis signaling pathways in major bone-associated cells.

Once the above research results are tested in clinical trials, they can not only alleviate bone structural abnormalities by inhibiting ferroptosis, but also effectively delay OA and OP, prevent the spread of OS, and then improve the motor dysfunction of patients.

In conclusion, a deep understanding of the scientific significance of ferroptosis in bone-related cells, the core regulation of ferroptosis, and the design of targeted clinical trials will contribute to the precise treatment of multiple orthopedic diseases.

## Data Availability

Not applicable.
